# Title: pancreatic‐type mixed acinar neuroendocrine carcinoma of the stomach: a case report and review of the literature

**DOI:** 10.1186/s13000-021-01070-x

**Published:** 2021-02-02

**Authors:** Yuka Ooe, Kishichiro Watanabe, Isaya Hashimoto, Satoshi Takenaka, Toshihiko Ojima, Seiichi Yamamoto, Hisatake Fujii

**Affiliations:** 1 Department of Surgery, Toyama Nishi General Hospital, 1019 Shimokutsuwada, Fuchumachi, Toyama, Toyama 939-2716 Japan; 2 Watanabe’s Consultancy for Pathological Diagnosis, 1007 Surpass Sakurada-cho Ichibankan 3-30-1 Sakurada-cho, Kanazawa, Ishikawa 920-0057 Japan; 3 Department of Surgery, Suzu General Hospital, 1-1 Nonoemachi, Suzu, Ishikawa 927-1213 Japan

**Keywords:** Gastric cancer, Acinar cell carcinoma, Neuroendocrine tumor, MiNEN, Laparoscopic surgery

## Abstract

**Background:**

The majority of gastrointestinal tumors are adenocarcinomas. Rarely, there are other types of tumors, such as acinar cell carcinoma, and these are often called pancreatic-type acinar cell carcinomas. Among these tumors, some are differentiated into neuroendocrine components. A few of them are MiNENs.

**Case presentation:**

The patient was an 80-year-old male who was referred to our hospital for treatment of a pedunculated gastric tumor. It was 5 cm in diameter and detected in the upper gastric body with upper GI endoscopy conducted to investigate anemia. In the biopsy, although hyperplasia of gastric gland cells was noted, no tumor cells were found. Retrospectively, the diagnosis was misdiagnosed. An operation was arranged because bleeding from the tumor was suspected as a cause of anemia and because surgical resection was considered to be desirable for accurate diagnosis. Hence, laparoscopic and endoscopic cooperative surgery was performed. In the pathological examination, several types of epithelial cells that proliferated in the area between the mucosa and deep inside the submucosa were observed. These consisted of acinar-glandular/trabecular patterns and solid. A diagnosis of pancreatic-type acinar cell carcinoma of the stomach with NET G2 and G3 was made based on characteristic cellular findings and the results of immunostaining tests. Each of them consisted of more than 30% of the lesion; a diagnosis of pancreatic-type mixed acinar neuroendocrine carcinoma (pancreatic-type MiNEN) of the stomach or a type of gastric MiNEN was obtained. Anemia was resolved after the operation, and the patient was discharged from the hospital without perioperative complications.

**Conclusions:**

Pancreatic-type ACC of the stomach that is differentiated into neuroendocrine tumors is very rare. Hence, we report this case along with a literature review.

## Background

Acinar cell carcinoma (ACC) is a rare type of tumor that makes up 1–2% of pancreatic tumors [1]. In 2002, Fukunaga [2] became the first to report a case of ACC that developed in the stomach. Since then, several cases of pancreatic-type acinar cell carcinoma of the stomach have been reported. The term “pancreatic-type acinar cell carcinoma”, which Sun et al. [3] first used in a case of gastric cancer, has also been used for cancers of gastrointestinal and hepatobiliary locations other than the stomach.

In 2019, the WHO classification of neuroendocrine neoplasms was modified; mixed neuroendocrine-nonneuroendocrine neoplasms (MiNENs) are mixed epithelial neoplasms in which a neuroendocrine component is combined with a nonneuroendocrine component, each of which is morphologically and immunohistochemically recognizable as a discrete component and constitutes > 30% of the neoplasm [4]. ACCs that develop in extrapancreatic tissues and MiNENs are so rare that their characteristics are not yet well understood.

We report here a case of pancreatic-type mixed acinar neuroendocrine carcinoma (pancreatic-type MiNEN) of the stomach (gastric MiNEN) with a literature review.

## Case presentation

The patient was an 80-year-old male who had been followed up for hypertension and dyslipidemia. His height was 167 cm, his weight was 69.7 kg, and he had a surgical history of appendectomy for appendicitis. Anemia was observed in the conjunctiva. The abdomen was flat and soft without tenderness. There was a surgical scar from the appendectomy. No superficial lymph node was palpated.

**Fig. 1:**
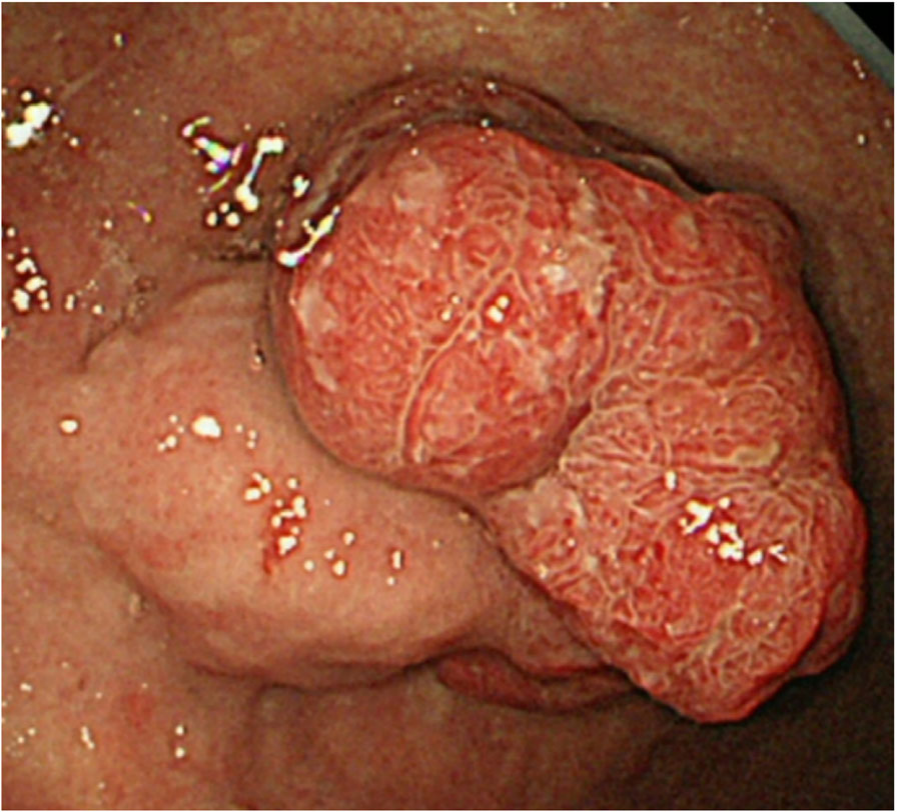
Esophagogastroduodenoscopy findings. A large ulcerated fungated mass in the gastric upper body

In the blood test, the Hb level was 10.2, suggesting anemia. AMY at 66 IU/L was within the reference range, and no abnormality in other blood biochemistry tests was observed. Tumor marker levels were CEA 1.9 ng/mL and CA19–9 13.6 U/mL, both within their reference ranges. A pedunculated gastric tumor was detected in the upper gastric body with upper gastrointestinal endoscopy (Fig. 1). Reddish changes, which may indicate epithelial tumors, were detected on the apex of the tumor. Ulcer formation was observed, and invasion to the submucosal tissues could not be ruled out. There was no obvious bleeding detected in this examination. A biopsy was performed, and histopathological malignancy was not revealed. A kind of gastric gland cell hyperplasia was diagnosed, probably because it seemed that no significant atypia was found in the specimen.

**Fig. 2:**
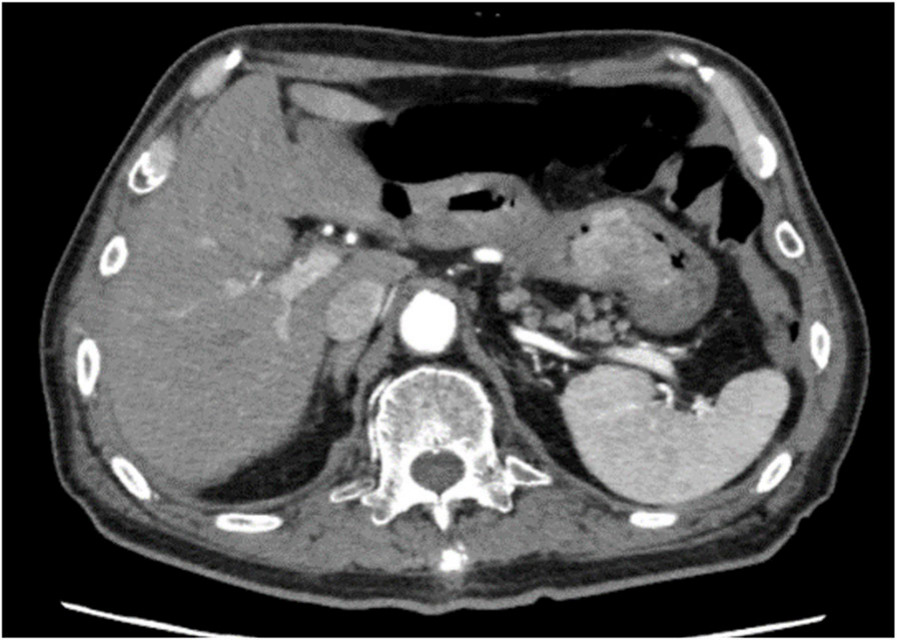
Abdominal computed tomography. Enhanced gastric tumor incarcerated to the antrum

On abdominal/pelvic computed tomography (Fig. 2), a neoplastic lesion of 5 cm in diameter was observed in the upper gastric body. As it was located on the pyloric side of the upper gastric body, pyloric obstruction was also suspected. There was no finding suggesting a pancreatic tumor, lymphadenopathy, or metastatic lesions. No ascites was observed.

A surgical procedure was arranged for the following reasons: bleeding from the tumor was suspected as a cause of anemia, ball valve syndrome had been induced by the tumor, the possibility of malignancy could not be ruled out, and the risk of incomplete resection or bleeding with endoscopic resection was considered high.

Laparoscopic endoscopic cooperative surgery was performed. A tumor incarcerated from the greater curvature of the upper gastric body to the duodenum was guided into the gastric cavity under endoscopic observation. Full-thickness resection was performed while maintaining an approximately 10-mm margin. The resected site was closed with layer-to-layer sutures.

**Fig. 3:**
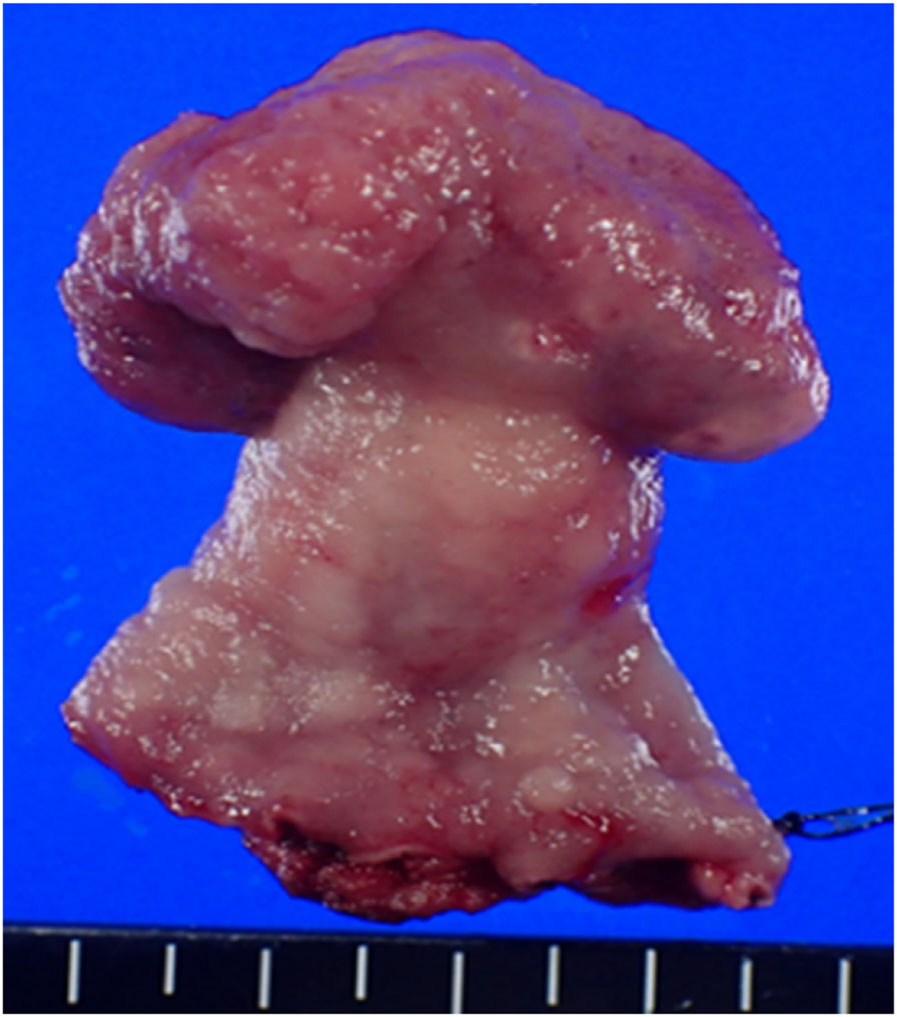
Macroscopic findings

**Fig. 4:**
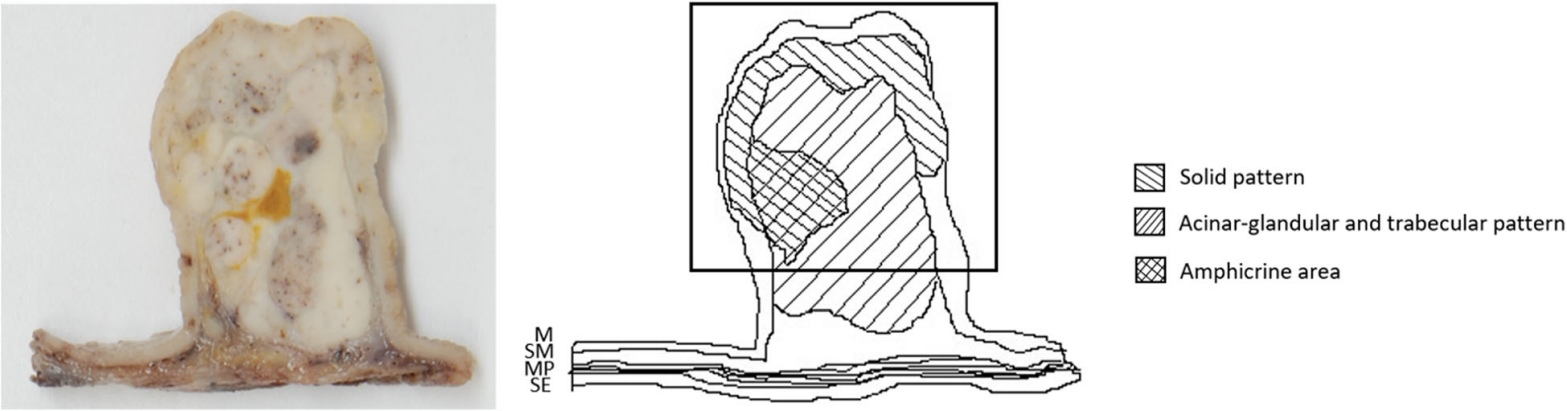
Schematic diagram of each component. The square indicates the area shown in Fig. 6

Macroscopically, an elastic-hard grayish-white solid neoplastic 3.5 × 3.3 × 2.5-cm lesion protruding inside the gastric cavity was observed. Some parts were edematous on the sectional surface, and no necrosis or bleeding was observed (Figs. 3, 4).

**Fig. 5:**
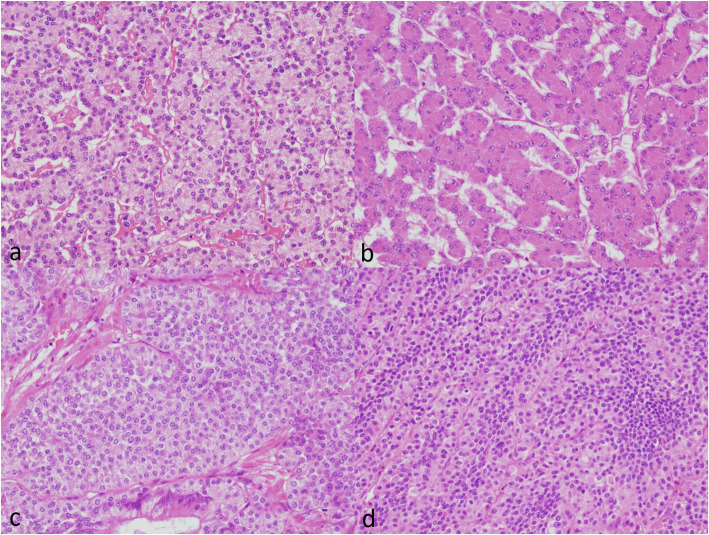
(**a**, **b**, **c**, **d**) **a**: acinar-glandular pattern × 200 **b**: trabecular pattern × 200 **c**: solid pattern × 200 **d**: solid pattern (large and small cells) × 200

Whole-section slides from the resected tissues were made. Histologically, the lesion consisted of a remarkable proliferation of several types of epithelial cells in the mucosa and submucosa. The border of the lesion was very irregular in the mucosa or the superficial layer of the lesion; however, it was clear in the submucosa or middle and deep inside layer of the lesion with partial fibrosis and a capsule. The propria muscularis was not involved. Proliferating epithelial cells showed basically acinar-glandular, trabecular and solid patterns (Fig. 5). These patterns were seen separately in some parts and intermingled with one another in other parts. Small amounts of stromal mucus deposition were found in some parts of the superficial layer of the lesion. Regarding the general tendency, the solid pattern was found mainly in the mucosa or the superficial layer of the lesion, while the acinar-glandular and trabecular patterns were observed in the submucosa or deep inside of the lesion. Since the morphological atypia of these proliferative epithelial cells was not significant and these cells resembled pancreatic acinar cells well, the possibility of a special type of ectopic pancreas was considered first. However, normal pancreatic cells (e.g., acinar, ductal, islet cells) that support a diagnosis of heterotopic pancreas were not observed at all.

The cells that proliferated as acinar-glandular and trabecular patterns (Fig. 5a and b) contained eccentric nuclei, which appeared to be slightly enlarged, presenting conspicuous nucleoli, as well as eosinophilic granules inside the cells. The cells that proliferated as the solid pattern (Fig. 5c) had oval nuclei, presenting salt and pepper-like chromatin that was distributed heterogeneously and slightly eosinophilic granules inside the cells. Aggregations/mixtures of small cells and large cells were also observed on an image (Fig. 5d). Although significant atypical cells were rare for each type, the characteristics of the nuclei and nucleoli of the cells mentioned above and irregularity of the border of the lesion in the mucosa or the superficial layer led us to the idea that the lesion may be acinar cell carcinoma with some neuroendocrine features.

**Fig. 6:**
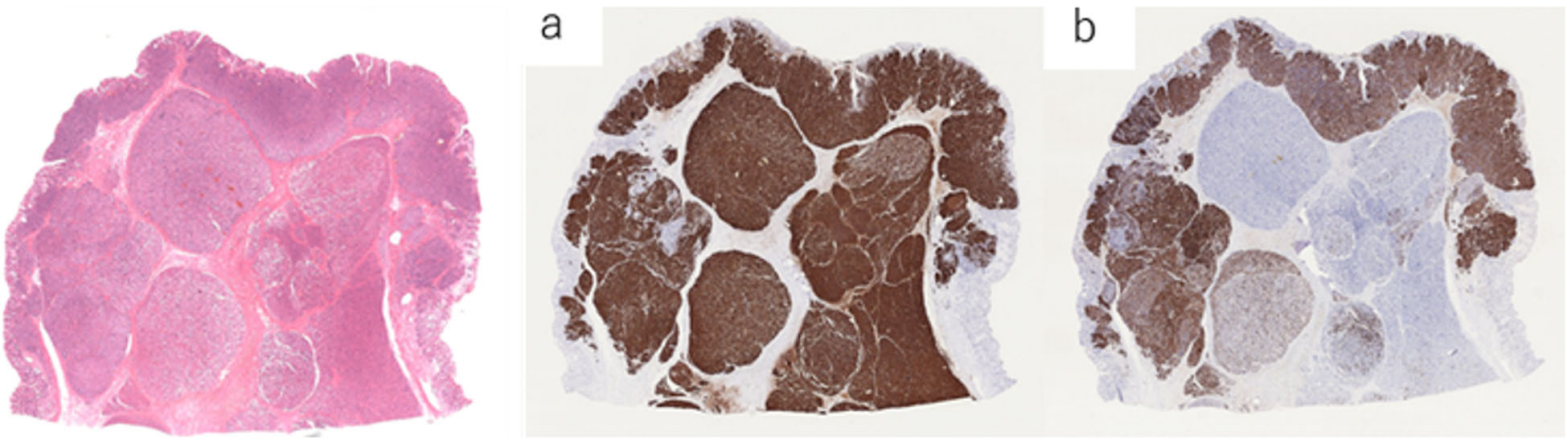
(**a**, **b**) **a**: anti-BCL-10 antibody staining **b**: chromogranin A staining

To confirm this idea, immunostaining was performed using various pancreas-related markers or neuroendocrine markers. Immunostaining tests revealed that most of the lesion was strongly positive for BCL-10 antibody (Fig. 6a) and trypsin but negative or weak in some small parts of the superficial layer of the lesion. In addition, strongly positive results were obtained for chromogranin A, synaptophysin, and CD56 neuroendocrine markers, especially in the superficial layer of the lesion, in which the negative or weak parts for BCL-10 and trypsin were almost positive. Regarding neuroendocrine reactivity in this case, chromogranin A was most remarkable among the three neuroendocrine markers, suggesting that it accounted for 40% of the lesion (Fig. 6b). Concerning the mitotic rate and Ki-67 index of the lesion, the proportions of acinar-glandular and trabecular cells proliferating mainly in the submucosa or middle and inside of the lesion were < 2 and < 3%, respectively, while those of solid cells proliferating mostly in the mucosa or superficial layer of the lesion were mainly 2–5 and 3–20%, respectively. Moreover, in some small parts of the layer, the proportions were > 20 and 20–40%. Epithelial markers such as CK7, CK20, CAM5.2 and AE1/AE3 were positive in the entire lesion as well as in the existing gastric mucosal epithelium. CK19, which is a pancreatic ductal marker, showed positivity in some parts of the superficial layer of the lesion and the existing gastric mucosa but negativity in the middle or deep inside of the lesion.

Additionally, the tests for pancreatic and digestive tract-related hormones (e.g., insulin and gastrin) were negative. No intranuclear positive finding was observed with beta-catenin either. The immunostaining tests are presented in Table 1.

**Table 1:** Antibodies employed in the immunohistochemical study and results

	Growth pattern					
Antibody	Acinar-glandular pattern	Solid pattern	Overlying gastric mucosa	Clone	Source	Dilution
BCL-10	+++	++	–	331.3	Santa Cruz Biotechnology	1:50
Trypsin	+++	++	–		Merck Millipore	1:2000
Chromogranin A	+	+++	–	SP12	Thermo Fisher Scientific	1:75
Synaptophysin	+	++	–	SP11	Thermo Fisher Scientific	1:75
CD56	+	++	–		Nichirei Bioscience	prediluted
Amylase	+	+	+			
Insulin	–	–	–		DakoCytomation	1:100
Glucagon	–	–	–		DakoCytomation	1:200
Gastrin	–	–	–		DakoCytomation	1:300
Serotonin	–	–	–		DakoCytomation	1:50
Somatostatin	–	–	–		Roche	prediluted
Pancreatic polypeptide	–	–	–		DakoCytomation	1:600
β-catenin	+ (cell membrane, cytoplasm)				DakoCytomation	1:200
CK7	–	–	+	OV-TL12/30	DakoCytomation	1:75
CK19	–	–	+	RCK108	DakoCytomation	1:75
CK20	–	–	+	KS20.8	DakoCytomation	1:75
CAM5.2	+	+	+		Becton, Dickinson and Company	prediluted
CKAE1/AE3	+	+	+		DakoCytomation	prediluted

The nearest resected margin from the lesion was 0.5 cm and free from the tumor. No fundic gland was observed in the existing gastric mucosa. The mucosa consisted of crypt epithelium with intestinal metaplasia and hyperplasia, and no obvious malignancy was detected.

Regarding the tumor tissues presenting acinar-glandular and trabecular patterns, a diagnosis of pancreatic-type acinar cell carcinoma of the stomach was made according to the characteristic findings, such as clear and swollen nucleoli and the results of immunostaining tests. Most of the tumor tissue that presented a solid pattern showed neuroendocrine tumor (NET) G2 characteristics as well as acinar cell carcinoma. However, some parts of that area showed the characteristics of NETG3 without those of ACC.

Considering that each component accounted for more than 30% and that both of them had been mixed and shifted with each other, a diagnosis of pancreatic-type mixed acinar neuroendocrine carcinoma (pancreatic-type MiNEN) of the stomach (gastric MiNEN) was made.

Postoperative course: No perioperative complications were observed; the patient started consuming meals on day 3 after the operation, and on day 13, he was discharged from the hospital. In the blood test, the Hb level had increased to 14.1, suggesting that his anemia had been resolved. Unfortunately, the patient died of brainstem hemorrhage 4 months after the operation. No autopsy was performed.

## Discussion

In 2002, Fukunaga [2] became the first to report a case of acinar cell carcinoma that developed in the stomach. Since then, a total of 13 cases of gastric ACCs (including ACCs that also contain neuroendocrine tumor cells) have been reported [3, 5–12]. In addition, there are other reports of ACCs in the duodenum, papilla, small intestine and liver; ACCs that develop outside the pancreas are often called pancreatic-type ACCs [13–17].

Rarely, some gastrointestinal tumors demonstrate both exocrine and neuroendocrine differentiation. According to the World Health Organization (WHO) 2010 classification [18], this group of tumors are called mixed adenoneuroendocrine carcinomas (MANECs), and in the 2017–2019 WHO classification [4, 19], these tumors were renamed mixed neuroendocrine-nonneuroendocrine neoplasms (MiNENs). Recently, La Rosa et al. [20, 21] advocated a detailed classification (Table 2).

**Table 2:** Classification of MiNEN according to their grade of malignancy

	Non-neuroendocrine component	Neuroendocrine component
High-grade MiNEN	Carcinoma^a^ or adenoma	Poorly differentiated neuroendocrine carcinoma (G3) of small- or large-cell type
Intermediate-grade MiNEN	Carcinoma^a^	Well-differentiated neuroendocrine tumour (G1-G2)
	Amphicrine carcinomas	
Low-grade MiNEN	Adenoma	Well-differentiated neuroendocrine tumour (G1-G2)

Adapted from La Rosa et al [7]. a Carcinoma generally consists of adenocarcinoma but can be squamous-cell or acinar-cell carcinoma as well

It is difficult to obtain a diagnosis of pancreatic-type ACCs of the stomach with endoscopic biopsy [11], and it seems even more difficult to diagnose MiNENs because it seems that the terms and concepts of “pancreatic-type ACC”, “pancreatic-type MiNENs” and “gastric MiNEN” are not widely understood and accepted. Chymotrypsin and amylase markers can be useful in these diagnoses [11]. It has been reported recently that immunostaining with anti-BCL-10 antibody can be a marker that is high in terms of both sensitivity and specificity for pancreatic acinar cells as well as for cells that present acinar cell differentiation [22]. For this patient, who had been diagnosed with gastric gland cell hyperplasia by endoscopic biopsy before the operation, the result of a postoperative immunostaining test was strongly positive for anti-BCL-10 and chromogranin A antibodies. Hence, immunostaining tests using anti-BCL-10 antibody and other markers can be useful and should be considered for preoperative diagnosis.

It has also been reported that 42% of ACCs of the pancreas would be positive for a neuroendocrine marker [8]. Therefore, there is some confusion between the diagnosis of acinar cell carcinoma and neuroendocrine carcinoma of the pancreas. In general, a solid pattern is morphologically considered to be one of the acinar cell carcinoma patterns of the pancreas [4]. However, it seems valid that the solid pattern in our case can be regarded as a morphologically recognizable discrete component, which is consistent with the definition of MiNEN.

Our case is very rare in that it was a type of gastric MiNEN that consisted of pancreatic-type ACC and neuroendocrine tumor that was a mixed acinar neuroendocrine carcinoma or pancreatic-type MiNEN of the stomach. In addition, a small amount of stromal mucus deposition and some CK19-positive cells were detected in the superficial layer of the lesion in our case. These findings suggest that our case might be related to mixed acinar neuroendocrine ductal carcinoma.

To the best of our knowledge, 13 cases of gastric ACCs and gastric MiNENs accompanied by pancreatic acinar cell differentiation have been reported [2, 3, 5–12] (Table 3). Regarding the onset mechanism, it was considered that they developed from the ectopic pancreas and had been called “pancreatic-type ACC of the stomach” in some reports. Of these, ectopic pancreas was detected histologically in only one case [7]. In other cases, it was considered that the ectopic pancreas might have been destroyed and vanished due to tumor proliferation. Gastric MiNENs usually consist of well-differentiated adenocarcinoma and neuroendocrine carcinoma (NEC) components [23], and there is a tendency in the conditions of their distributions, namely, that the latter component often exists deep inside the lesion and the former in the surface of the lesion [24]. Regarding the onset mechanism of MiNENs (MANECs), Makuuchi et al. [24] proposed that during the process in which adenocarcinoma, which originally develops within the mucosa, invades deep inside the lesion, neoplastic endocrine cells that have higher proliferation potency would grow; hence, NEC is observed following MANEC. However, an opposite tendency was observed in the present case. In other words, neuroendocrine tumors existed on the surface of the lesion, and the ACC was located deep inside the lesion. As an ectopic pancreas is commonly detected in the submucosa [25], it can be considered that these distributions may support the idea that they develop from an ectopic pancreas. Regarding the site of onset, an ectopic pancreas in general is likely to develop on the pyloric side [26]. However, in previous reports, it developed in various sites other than the pyloric side, such as near the cardiac orifice or in the upper gastric body. In the present case, it also developed in the upper gastric body. Metastasis of tumors from other organs, such as the pancreas, could be ruled out for this patient, as there was no significant finding on imaging that indicated a primary lesion in this case. Hence, we consider the following 3 conditions, other than development from the ectopic pancreas, as the onset mechanism of this tumor: 1. A tumor that simultaneously contains two distinctive cellular components develops, 2. A tumor that contains two components originating from a pluripotent stem cell with the potential for divergent differentiation, and 3. A condition in which part of the nonneuroendocrine tumor is turned into a neuroendocrine tumor or in which part of the neuroendocrine tumor is turned into a non-neuroendocrine tumor. The immunostaining study with the most surface layer of the lesion indicating a neuroendocrine neoplasm revealed partial positive BCL-10 staining. That may suggest the third condition.

**Table 3:** List of carcinomas with pancreatic acinar cell differentiation reported in the literature

Author, year	Age	Sex	Site	Diagnosis	Metastasis	Ectopic pancreas	Therapeutic option	Adjuvant therapy	Outcome
Fukunaga, 2002 [2]	77	F	Fundus	MAEC	None	None	EMR	–	Alive, 7mo
Sun et al., 2004 [3]	86	F	Antrum	ACC	None	None	partial gastrectomy	–	NM
Jain et al., 2005 [5]	41	F	Body	MAEC	LN	None	partial gastrectomy, partial omentectomy	–	Alive, 2 yr
	61	M	Fundus	MAEC	LN, Liver	None	subtotal gastrectomy	–	Dead, 6mo
	72	M	Body	MAEC	None	None	subtotal gastrectomy	–	Dead, 4mo
Mizuno et al., 2007 [6]	73	M	Pylorus	NA^a^	LN, Liver	None	pancreaticoduodenectomy	Gemcitabine+Cisplatin+ 5-FU	Alive, 11mo
Ambrosini-Spaltro et al., 2009 [7]	52	M	Antrum	ACC	None	Presence	subtotal gastrectomy, omentectomy, cholecystectomy	NM	NM
Kusafuka et al., 2009 [8]	56	F	Body	MAEC	Peritoneum, Liver, Skin	None	total gastrectomy, splenectomy, right-half colonectomy	S-1 + Cisplatin	Dead
Coyne, 2012 [9]	77	F	Fundus	ACC	None	None	partial gastrectomy	–	Dead, 1mo
Yonenaga et al., 2016 [10]	67	M	Body	MAEC	LN	None	distal gastrectomy, lymph node dissection	S-1 monotherapy	Alive, 21mo
	63	M	Antrum	ACC	Liver	None	–	Gemcitabine+Erlotinib	Dead, 5mo
Kim et al., 2017 [11]	54	M	Cardia	ACC	None	None	laparoscopic wedge resection	–	Alive, 33mo
Fujita et al., 2019 [12]	67	M	NM	MiNEN	None	None	ESD	–	Alive, 15mo

*NM* not mentioned

*ACC* acinar cell carcinoma

*MAEC* mixed acinar endocrine carcinoma

*MiNEN* mixed neuroendocrine-non-neuroendocrine neoplasms

*EMR* endoscopic mucosal resection

*ESD* endoscopic submucosal dissection

*LN* lymph nodes

*mo* months

*yr* years

NA^a^, the immunohistochemistry for neuroendocrine markers was not performed

Gastric MiNENs usually consist of adenocarcinoma and NEC components. In such a case, it is assumed that the latter component would determine the prognosis of MiNENs [27].

According to a report on pancreatic tumors, the prognosis of ACCs is better than that of ductal adenocarcinomas, which account for most pancreatic tumors. However, it is still challenging, as the median survival time is approximately 19 months and the 5-year survival rate is approximately 25%. Malignant findings such as significant atypical cells or frequent mitotic cells were rare in the present case. The effects of such morphological characteristics, the immunostaining test results, and molecular abnormalities on disease prognosis have been investigated in recent studies. However, only tumor staging has ever been verified as a factor associated with prognosis [28].

As there is no standard opinion for the treatment of gastric MiNENs, it is considered important to determine a treatment approach for each case according to the tumor components. A management approach for gastric ACCs has not yet been determined, as the number of cases is limited. However, surgical treatment should be the first option for localized pancreatic ACCs [29]. According to a report on 865 cases studied based on the National Clinical Database, satisfactory outcomes were achieved in the surgically treated group compared to the nonsurgically treated group; the five-year survival rates were 36.4% (median, 27 months) and 10.4% (median, 7.1 months), respectively. For unresectable or distant metastasis cases, chemotherapy or radiotherapy for downstaging purposes could be an option to downstage the disease to allow for surgical resection [30]. In the present case, the outcome was death due to brainstem hemorrhage. However, it was considered to be an accident, as the relationships between MiNEN, ACC, NET/NEC and cerebrovascular diseases are currently unknown.

## Conclusion

We experienced a case of pancreatic-type ACC of the stomach. A diagnosis of MiNEN was also made, as nearly 40% of the tumor cells had been differentiated into neuroendocrine tumor cells (pancreatic-type mixed acinar neuroendocrine carcinoma or pancreatic-type MiNEN). The number of reports on pancreatic-type ACCs or MiNENs has been increasing due to advanced immunological technologies. However, these are not yet well understood, as the number of cases is still very small. Further studies are required to clarify the characteristics of the tumor, to determine the treatment approach and to improve patient prognosis.

## Data Availability

The dataset supporting the findings and conclusions of this case report is included within this article.

## Abbreviations

ACC: Acinar cell carcinoma
MiNEN: Mixed neuroendocrine-nonneuroendocrine neoplasms
NET: Neuroendocrine tumor
WHO: World Health Organization
MANEC: Mixed adenoneuroendocrine carcinoma
NEC: Neuroendocrine carcinoma
